# A novel framework for operationalising patient and public involvement: lessons from designing an AI-informed exercise prescription grant

**DOI:** 10.1186/s40900-025-00801-4

**Published:** 2025-10-29

**Authors:** Lucy Smith, Jacob Keast, Jo Flowers, Hajira Dambha-Miller

**Affiliations:** 1 Primary Care Research Centre, University of Southampton, Southampton, UK; 2 Public contributor, Southampton, UK

**Keywords:** Patient and public involvement, Healthcare research, Physical activity and chronic illness

## Abstract

**Background:**

While grant funders such as the National Institute of Health and Care Research increasingly require patient and public involvement (PPI) across all stages of grant development, there remains limited documentation on how meaningful PPI involvement is operationalised, and how its impact is measured. This aim of this case study was to describe how PPI can be embedded in a research grant application to develop an Artificial Intelligence-informed exercise programme for individuals living with chronic illness.

**Methods:**

We constructed a four-stage framework to operationalise the process of integrating PPI across the grant development process, including: gathering initial PPI feedback, establishing a lived experience advisory panel (LEAP), co-developing the grant submission, and conducting an impact assessment guided by the 2019 UK Standards for Public Involvement, GRIPP2 reporting checklist, and prior PPI expertise.

**Results:**

Our framework for incorporating PPI across the grant development process was found to be impactful across all measures of the UK Standards for Public Involvement. Following stage 1, we implemented a sequential process for forming a lived experience advisory panel (stage 2) and demonstrated a robust, approach to document the impact of PPI on grant development (stage 4). Important themes that developed from the group feedback during stage 1 and 2 included concerns over trust and consistency of physical activity recommendations, empowerment through active engagement, meeting user needs, appropriateness of data collection methods, digital literacy and widening recruitment opportunities which influenced the grant design. Significant learnings from stage 3 and 4 for future PPI highlight the importance of considering wider contextual factors which influence the impact of PPI on grant development. Factors such as engaging with local community groups serving under-represented patient cohorts to increase the diversity of PPI participants, and consideration of PPI activity accessibility for those with chronic health conditions.

**Conclusions:**

This paper provides a novel framework which can be used by researchers to plan and carry out meaningful PPI in their grant applications. Embedding PPI across the grant development process resulted in meaningful contributions that shaped the proposal and will continue to influence the project’s trajectory. This paper recommends employing a clear methodology for involving PPI and capturing its impact from the outset to enhance the relevance, transparency, and inclusivity of research. This case study highlights the value of sustained and structured PPI in co-producing impactful health research and grant activities.

**Supplementary Information:**

The online version contains supplementary material available at 10.1186/s40900-025-00801-4.

## Abstract (plain language summary)

This paper describes a new framework to guide how people and patients can be involved in shaping a research grant application. In this example, we show how people with lived experience of chronic illness were involved in all stages of shaping a research grant. The grant aimed to explore the use of artificial intelligence (AI) to help create personalised exercise plans in general practice.

We asked for feedback and ideas from people with chronic illness, and their carers, at four key stages of the grant-writing process. We ran a face-to-face workshop with seven people, from which four participants agreed to join a lived experience advisory panel (LEAP). This panel continued to be involved as the project progressed, reviewing drafts of the grant application as it was written. Their input helped to shape research questions and design, improve the details of the proposal, and ensure the research was relevant and inclusive to what the public wants. This paper provides a new method for researchers to plan and carry out meaningful patient and public involvement to improve grant applications, and ensure future research projects are relevant for the people who will use them, leading to more practical and beneficial outcomes for patients and the public.

**Supplementary Information:**

The online version contains supplementary material available at 10.1186/s40900-025-00801-4.

## Background

As defined by the Health Research Authority (HRA), patient and public involvement (PPI) is research carried out *“with” or “by”* members of the public, rather than *“to”*,* “for”* or *“about”* them [1]. This is distinct from public engagement, which refers to sharing research findings with the public. The benefits of PPI during the design phase are well established [2]. Involving people with lived experience early can help ensure that research questions are relevant, materials are understandable, and studies are more accessible [3]. This increases the potential for successful knowledge mobilisation at later stages. These benefits are significant for research in complex or emerging fields, such as the use of artificial intelligence (AI) in healthcare [4]. In this case study, the research grant focused on AI-based exercise prescriptions for people living with multiple long-term conditions (MLTC), which affects almost 15% of adults in the UK [5]. Public attitudes towards AI in healthcare are mixed—only a slight majority (54%) of patients report support for its use [6]. Including public contributors and lived experience from the outset of grant applications can help identify barriers to patient engagement early, and address concerns during the design stage [7]. Equally important is the inclusion of underrepresented groups in the development process [7]. People most affected by physical inactivity—often due to health inequalities—are also less likely to be involved in research [8]. Ensuring that their perspectives shape study design can improve accessibility, equity, and the real-world relevance of future interventions being developed.

The benefits of including PPI across the study design are now recognised on a national level across the UK. Funding bodies such as the National Institute for Health and Care Research (NIHR) require all applicants to demonstrate how PPI has been involved in designing grant proposals, and guidance for applicants on working with people and communities has been provided [9]. Tools such as the GRIPP2 reporting checklist, developed to improve transparency, consistency, and quality of PPI reporting activities, offer guidance on how to report PPI in health and social care research [10]. Further practical guidance is available to researchers through organisations such as Learning for Invovlement, Cancer Research UK, or the NIHRs Centre for Engagement and Dissemination and NIHR PenARC.

Despite these resources, there is still a paucity of research documenting how PPI is carried out practically to support each stage of grant development – from conception, to writing, and evaluation before submission. Few prior studies explicitly describe how PPI has been used to inform and shape a funding application, and to date only a few studied have explored practical PPI frameworks for researchers to follow, leading to inconsistency in methods or approach [11]. Moreover, much of previous PPI literature lacks empirical analysis of impact, relying instead on limited or anecdotal evidence, making it difficult to understand how PPI has impacted both the research and the people involved [12]. These gaps limit opportunities for shared learning, making it harder to build a collective understanding of best practice and impact in this area [2]. Culminating in a lack of clarity around how PPI should work, and how to ensure it is meaningful rather than tokenistic, which is frequently cited as a barrier to implementation [2–4, 7].

This aim of this case study was to describe how PPI can be embedded in a research grant application to develop an Artificial Intelligence-informed exercise programme for individuals living with chronic illness. In this paper, we outline how a four-stage framework was developed to involve patients and members of the public in shaping a research grant focused on AI-based exercise prescriptions in chronic illness. We describe the methods used to embed and measure the impact of PPI throughout the grant development process, from initial idea generation through to final proposal submission. Rather than reporting on a research study, this paper aims to document the framework we used to implement PPI in the grant development process. It assesses the impact of this process to influence the design of a future grant application, to share practical learning points, and to contribute to the limited evidence base on PPI involvement at the grant application stage.

## Methods

**Fig. 1:**
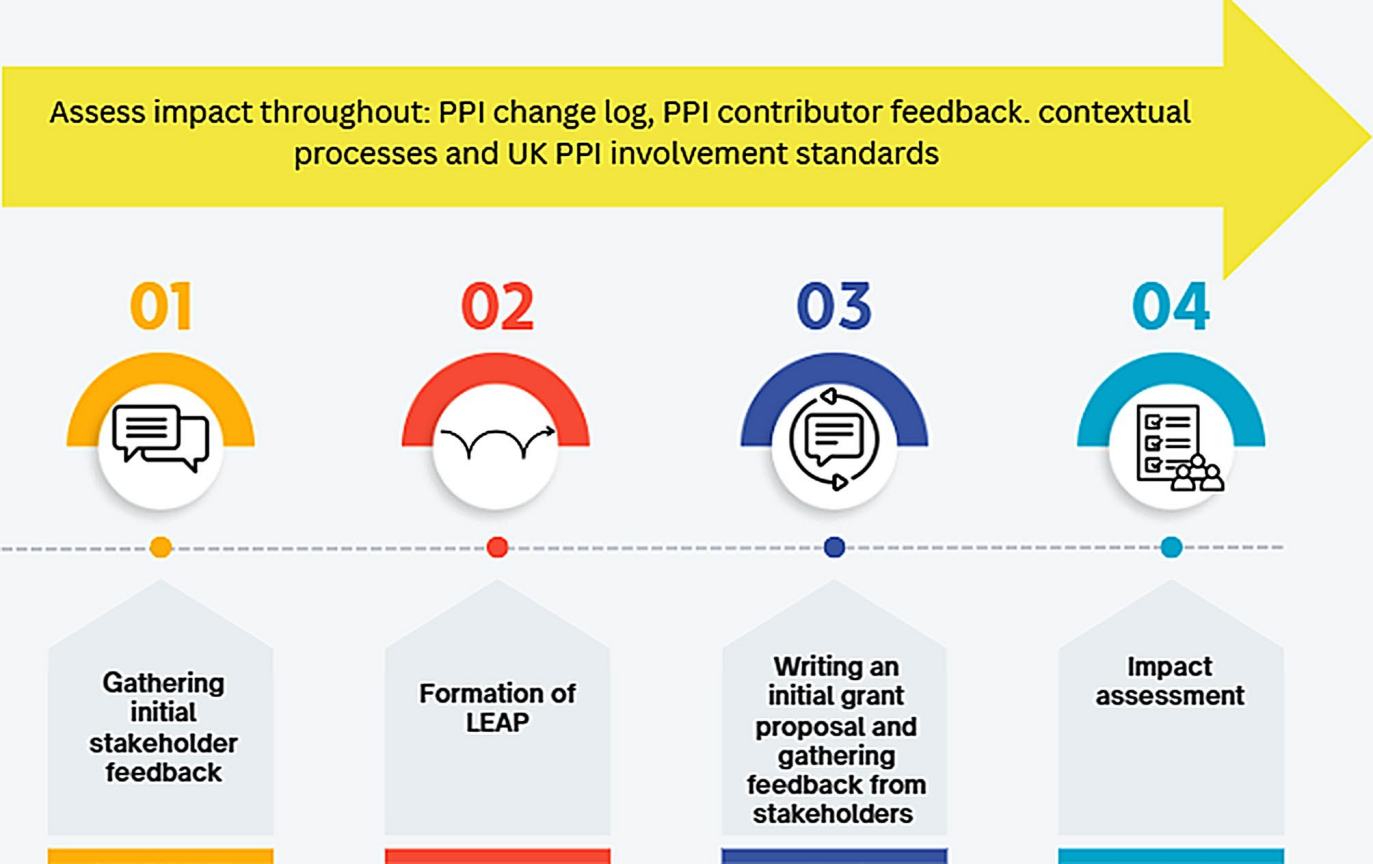
Diagramatical summary of PPI framework

We embedded PPI across four key stages of the grant development process, as outlined in Fig. 1; Table 1.

1. Gathering initial PPI feedback.
*Forming a lived experience advisory panel (LEAP group) for the grant.
3. Writing an initial grant proposal and gathering feedback from original PPI and LEAP groups on grant proposal.
4. Impact assessment.

*Please note, we acknowledge that this acronym overlaps with the ‘Learning, Evaluation, and Planning framework’ developed in Scotland for patient engagement work. In this paper, we use to refer to the formation of a group of contributors who have lived experience of MLTC, and/or being an informal caregiver.

The framework behind our approach was guided by the UK Standards for Public Involvement (2019) [13] and GRIPP2 reporting checklist [10]. Experiential guidance was also provided by LS, who has expertise in PPI and public engagement. Each stage of the PPI involvement framework used is described below.

### Stage 1: Gathering initial PPI feedback

The initial stage of PPI involvement consisted of a face-to-face workshop held in Oxford, England, to gather participant input to inform early development of the grant application. The workshop was designed to identify relevant research priorities, explore perceived challenges associated with digital health interventions, and assess the acceptability of AI technologies in the context of self-managed care. Recruitment targeted individuals with lived experience of chronic conditions and/or informal caregiving responsibilities. The event was promoted through regional and national PPI networks (see supplementary Fig. 1), as well as social media channels. Interested individuals were invited to register via a Microsoft Form hosted on a secure institutional platform, or by contacting the research team by email or telephone. No prior involvement in research was required.

All prospective participants received an information sheet, consent form, and a pre-event agenda outlining the key discussion topics. Individuals were asked to confirm attendance and share any accessibility requirements in advance of the event. Reasonable adjustments were made to ensure inclusive participation on the day, and regular rest breaks were included. Participants were reimbursed for travel expenses and received recognition for their contributions, in accordance with national guidance on PPI reimbursement [14].

The face-to-face session was facilitated by researchers experienced in PPI and qualitative engagement - LS, a public engagement research fellow, who led the session; and JK, a clinician and GP Exercise Referral Instructor with lived experience supporting patients exercise with long-term health conditions. A semi-structured discussion format was employed, guided by a topic framework. The workshop lasted two hours and followed an ‘ideas screening’ focus group approach, combined with a “listening café” to support informal but purposeful discussion during a shared lunch [15]. The concept of the ‘listening café’ incorporates eating as a group, taking part in a shared activity, and prompted questioning from the research team to provide a bridge for people who do not normally engage in research. VITOVA, an adaptive and AI-integrated exercise prescription software, was introduced as a case example for participants to visualise the potential of an exercise support software in practice. Field notes were taken during each activity, and the session was recorded, with written feedback collected from participants at the end via a Microsoft Form.

### Activity 1: Introduction and ideas screening

At the start of the session, participants were formally registered and briefed by members of the research team. They completed a consent form, image release form, and demographics questionnaire. The workshop began with introductions, including an icebreaker activity during which participants outlined their background and reasons for participating in the PPI process. LS presented an informational video, developed in collaboration with a public engagement coordinator, that summarised the challenges of maintaining exercise routines for individuals with chronic conditions. Participant feedback on the video and topic was invited through an open discussion. This was followed by a presentation from JK introducing VITOVA, an AI-driven digital tool used as an example of exercise prescription software. A structured question-and-answer session followed, during which LS recorded participant queries and concerns regarding the use of AI in healthcare. Participants were provided with focus group materials, including colour-coded cards (red = strong negative reaction, yellow = moderate reaction, green = strong positive reaction). Each participant selected a card to indicate their response to the concept of AI-supported physical activity tools. LS facilitated a structured discussion in which participants justified their selections, while JK recorded responses to support analysis.

### Activity 2 listening café

During the second activity, participants took part in a structured feedback session following the *Listening Café* format [15]. LS presented a draft of the plain English summary of the proposed research, including sections on the study aim, methodological approach, and anticipated impact. Participants were invited to provide feedback on each component. To support this, they were given pre-prepared feedback panels, which they completed and held up to prompt collective discussion. LS facilitated the group discussion, and JK documented participant responses and emerging themes.

Finally, participants were provided with a printed handout structured around four prompts: 1 - who should be recruited into the study; 2 - appropriate recruitment strategies; 3 - relevant outcome measures; and 4 - any additional feedback. These prompts were designed to inform core elements of research grant design and ensure alignment with the priorities and expectations of individuals with lived experience.

Following the workshop, participants received a summary document via email. This included a feedback form and revised plain English summary, documenting how their input had influenced proposed grant activities. Participants were invited to suggest further amendments to ensure their contributions were accurately represented and transparently integrated into the evolving research design.

### Stage 2: Forming a lived experience advisory panel (LEAP)

Workshop participants, and those who were unable to attend the in-person session, were invited to express interest in joining the LEAP, including the option to act as public co-applicants on the grant application. The requirements for LEAP participation reflected that of the PPI group: lived experience of, or being a carer for a person with long-term health conditions, and the capacity to engage with grant design and activity for the duration of the project where possible. Following the event, participants were contacted via email and asked to complete a Microsoft Form gathering feedback on their workshop experience and preference for LEAP participation. A virtual meeting was held to establish LEAP structure, roles, and remit. This process was guided by the panel’s overarching purpose: to ensure continuity of public involvement across all stages of the research project, beginning with the grant development phase. The LEAP was intended to contribute to several core activities, including:

- Refining research questions.
- Strengthening recruitment and engagement strategies.
- Reviewing participant-facing materials for accessibility and clarity.
- Advising on matters of equity, inclusion, and language.

LEAP members were engaged remotely via Microsoft Teams to maximise accessibility. LEAP contributors attended virtual meetings, and provided asynchronous feedback through follow-up emails and shared documents. This hybrid model enabled continuity of involvement across both in-person and remote formats, ensuring broader accessibility and sustained engagement throughout the grant development process.

**Fig. 2:**
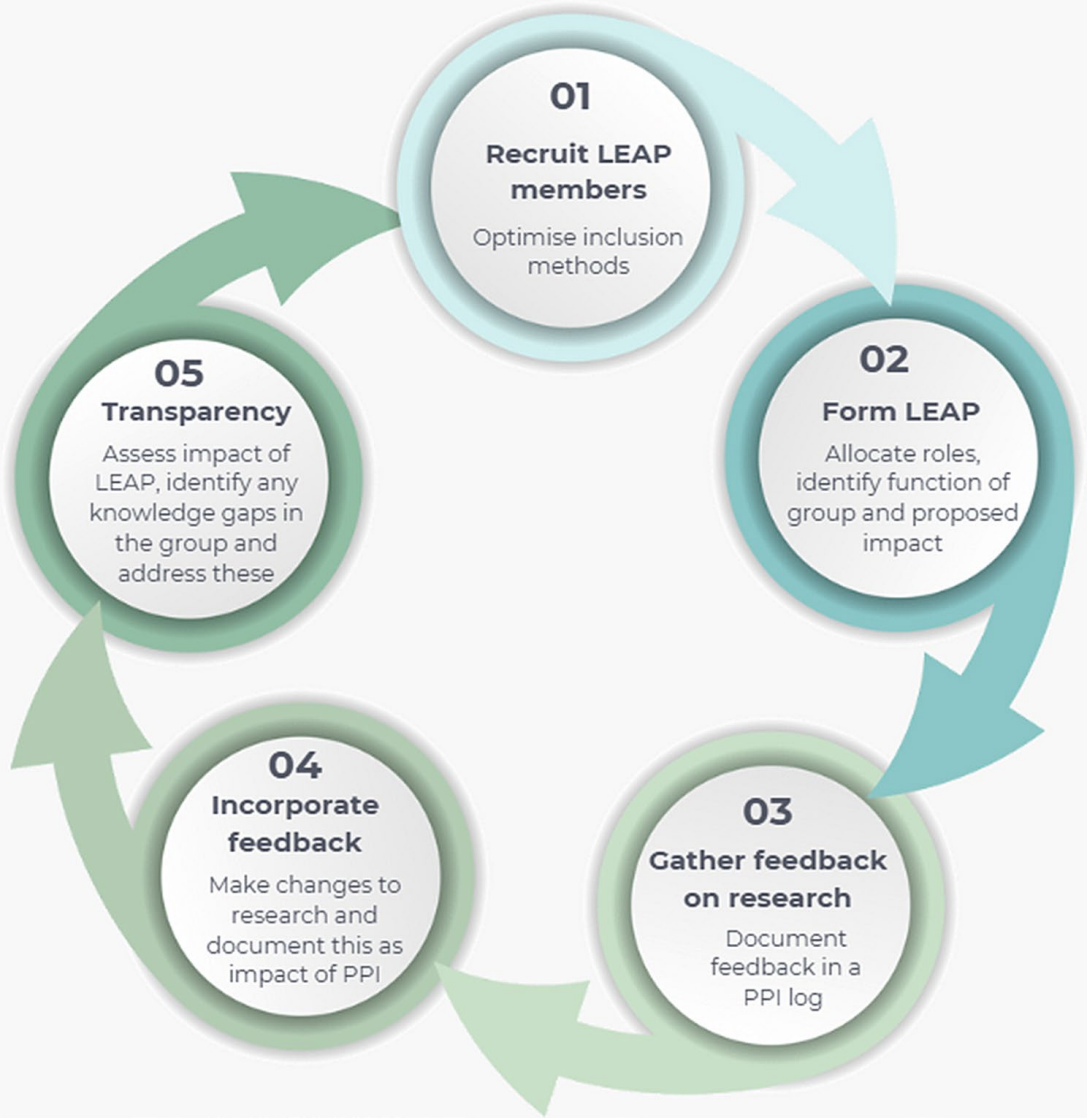
A visual representation of LEAP formation

PPI members were offered reimbursement for their time in accordance with NIHR guidance [14]. LEAP participants were invited to provide feedback on the clarity and relevance of the research question, the accessibility and inclusivity of the proposed methods, and the overall acceptability of the digital intervention. Input from the LEAP was relayed by LS to the broader research team and documented in the live PPI change log. Resulting changes to the proposal were tracked and incorporated into subsequent drafts of the grant application to ensure transparent integration of public feedback. Figure 2 provides a visual representation of the LEAP formation process, this diagram shows the formation is circular, not linear, taking into consideration the anticipation that the LEAP will evolve and more members may join the group in the future.

### Stage 3: Writing the initial grant proposal, and gathering PPI participant and LEAP feedback

Following the initial workshop, the research team met to review participant feedback. This input was synthesised into a structured table of changes, which informed the drafting of the initial research proposal. The early draft was developed in alignment with the themes identified during the workshop and was prepared for review by the LEAP members, who were invited to comment on the proposed aims, clarity of language, and consistency with previously gathered PPI input.

An updated draft of the application was shared with the LEAP for final review, which incorporated feedback from stage 1 and stage 2. Feedback was requested on the framing of research questions, inclusivity of the approach, and overall relevance to individuals living with chronic conditions. The research team incorporated this feedback into the final proposal, ensuring all recommendations were actioned or transparently addressed.

### Stage 4: Impact assessment

The purpose of the impact assessment was to document where PPI occurred in the grant development process, helping to evaluate how it influenced decision-making, improved research design, and supported inclusivity. Our assessment framework included the following components:

#### PPI change log

A live document was maintained to track all changes made to the proposal as a result of PPI input. Each entry records the original plan, PPI, contributor’s feedback, nature of the revision, and rationale for adopting or modifying the suggestion. The log was reviewed during research team meetings to ensure that PPI contributions were systematically considered and transparently actioned or addressed, feeding directly into the table of changes.

#### PPI contributor feedback

Structured feedback was collected from contributors through a short feedback form, and, where feasible, follow-up conversations. Contributors were asked whether they felt heard, whether their input had influenced grant design, as well as how accessible, inclusive, and respectful they found the process.

#### Contextual and process factors

Contextual and process factors surrounding PPI involvement were considered to reflect on the broader scope for encasing PPI in grant design. For example, factors such as the community being studied, the influence of the organisation leading the PPI, and experiences of the wider health and social care system will all set the ‘context’ of PPI. Process refers to the operational features which shaped the implementation of the PPI, including recruitment methods, accessibility of PPI activities, flexibility of PPI methods, and effectiveness of communication within the PPI process [16, 17].

#### UK standards for public involvement alignment review [18]

At the end of the development process, we assessed how our approach aligned with the UK Standards for Public Involvement [13]. Performance was assessed across the six domains: inclusive opportunities, working together, support and learning, communications, impact, and governance.

**Table 1:** Summary of PPI and LEAP engagement, focus, and format

Stage	Focus	Participants	Format
1	PPI grant design workshop	PPI participants	Synchronous: face to face session. Optional Microsoft forms mid-session for feedback.
1	PPI session pre-reading, agenda, consent form, feedback form post-session.	PPI participants	Asynchronous: PDF, Powerpoints, Microsoft Forms.
2	LEAP establishment	LEAP members	Synchronous Microsoft Teams session.
2	LEAP core activities and deliverables	LEAP members	Asynchronous: Email, shared documents, Microsoft forms.
3	Grant draft review	LEAP members	Asynchronous: shared documents, Microsoft forms.
4	PPI and LEAP contributor feedback for impact assessment	PPI and LEAP members	Asynchronous: Microsoft forms. Optional follow up conversatios.

## Results

### Stage 1: Gathering initial PPI feedback

#### Activity 1

A total of seven participants attended the in-person event, with demographic data provided in Table 2. Three key themes were developed from activity 1 group feedback: concerns over trust and consistency, empowerment through active engagement, and meeting user needs.

### Concerns over trust and consistency

Participant feedback on the research concept was positive. There was broad agreement that a digital platform for exercise prescription could be beneficial in supporting individuals with chronic conditions to engage in safe and effective physical activity. PPI contributors emphasised the need for such a tool to build user confidence in the safety and appropriateness of recommended exercises. Using a traffic light rating system, they were asked to indicate their initial response to the concept. Two selected green (strong positive reaction), four participants selected yellow (moderate), and one selected a red/yellow combination. The latter expressed specific concerns about the reliability of advice delivered via digital tools. This participant had previously used the Zoe health app and reported inconsistent guidance from different health coaches within the platform. In one instance, incorrect advice had exacerbated their symptoms. This experience led to broader concerns about the trustworthiness of digital recommendations and the need for transparency around how these are generated, including who delivers the advice and how quality is maintained.

Participants requested clarification on the role of a health coach within the proposed model. The research team explained that a health coach was envisioned as part of a social prescribing team. However, participants highlighted that access to such roles is highly variable across primary care settings, making it unlikely that this support would be consistent or equitable. Questions were also raised regarding how the platform’s algorithm would account for prescribed medications. After further explanation from the research team, participants reported that the concept was understandable and acceptable.

### Empowerment through active engagement

Empowerment and active engagement emerged as central themes. Participants expressed a preference for being involved in the exercise planning process, rather than receiving fully automated recommendations. They suggested that having access to a health coach for periodic check-ins, either by telephone or face-to-face, would enhance their sense of involvement and agency. The ability to customise exercise plans based on available equipment and physical capacity—such as selecting strength training options that do not require weights—was also viewed favourably. Initial responses to the use of artificial intelligence (AI) were mixed, primarily due to limited understanding of its function. After it was explained that AI would be used to adapt exercise plans over time by learning from user input, most participants considered the concept acceptable.

### Meeting user needs

Participants emphasised the need to include a broader range of chronic conditions within the platform, noting that the initial set appeared limited. Language choices were also identified as a concern. The term “compliant*”* was flagged as potentially stigmatising, particularly for individuals with mental health conditions. Participants felt that failure to comply with exercise recommendations could lead to feelings of guilt or disengagement. They recommended using more supportive, person-centred language. Flexibility and real-time adaptability of a digital tool were considered essential. They also expressed a strong desire for the platform to allow adjustments to their exercise plans based on daily symptom fluctuations—for example, being able to indicate that joint inflammation was particularly severe on a given day and receive modified guidance in response. Finally, participants supported the idea of integrating a peer community or forum feature to promote social support. However, some raised concerns about the potential for misinformation and the difficulty of moderating user content within an NHS-associated digital environment.

**Table 2:** Participant characteristics for those who attended the initial PPI workshop

Participant ID	Age	Gender	Profession	Highest Education level	Ethnicity	Lived experience
1	Not provided	M	Retired mechanical engineer	PhD	Asian	Informal carer
2	50	M	Police officer	HNC	White	Informal carer
3	46	F	Engineering	Degree	White	Patient (3 conditions, physical and mental)
4	64	F	IT	Degree	White	Patient and informal carer (6 physical conditions)
5	44	F	Accountant	Degree	White	Patient (2 physical conditions)
6	64	F	Organisational consultant	Master’s degree	White	Patient (8 conditions, physical and mental)
7	47	F	Dentist and researcher	Degree	Asian	Patient, (2 physical conditions)

#### Activity two

Participants reviewed the plain English summary of the proposed research, and broadly agreed that it communicated both the rationale for the study, and the planned methodology. Using a narrative synthesis three additional themes captured the overall feedback:

### Appropriateness of data collection methods

The use of the “think-aloud” method in the early stages of grant activity, involving small group discussion around a given task or topic, to promote further lived experience and healthcare professional insight was viewed positively. Participants noted that this would enable real-time expression of patient perspectives and support the inclusion of lived experience throughout the development process. The proposed study timeline was considered appropriate and realistic.

### Addressing digital literacy

The summary’s reference to addressing the digital divide—specifically by involving individuals with low digital literacy was positively received in principle. However, participants suggested removing this detail from the summary, as it would be difficult to measure whether this objective had been achieved. Instead, they recommended embedding this aim within the study’s design and processes rather than explicitly stating it as an outcome. To increase accessibility of the digital tool, there was detailed discussion on suggested delivery format, particularly the use of online versus in-person check ins. Participants agreed that both options should be offered to increase accessibility, particularly for individuals with limited digital skills, or accessibility barriers. A suggested solution to increase accessibility of the digital tool involved health coaches providing direct assistance with onboarding—using a shared tablet or laptop to guide individuals through setup and use of the platform. It was proposed that the platform generate printable exercise plans, which could be completed in hard copy and returned to the health coach during follow-up appointments, allowing updates to be entered into the system on the participant’s behalf.

### Widening recruitment opportunities

In relation to recruitment of participants from global majority communities, participants recommended proactive, community-based engagement strategies. One example included partnering with local religious or cultural leaders (e.g. an Imam at a mosque) and using community spaces for recruitment and data collection, rather than expecting individuals to attend unfamiliar academic or clinical research settings.

Participants also recommended ensuring diversity in the demographic of study participants within the grant and emphasised the need to support individual participant needs to reach this goal.

**Fig. 3:**
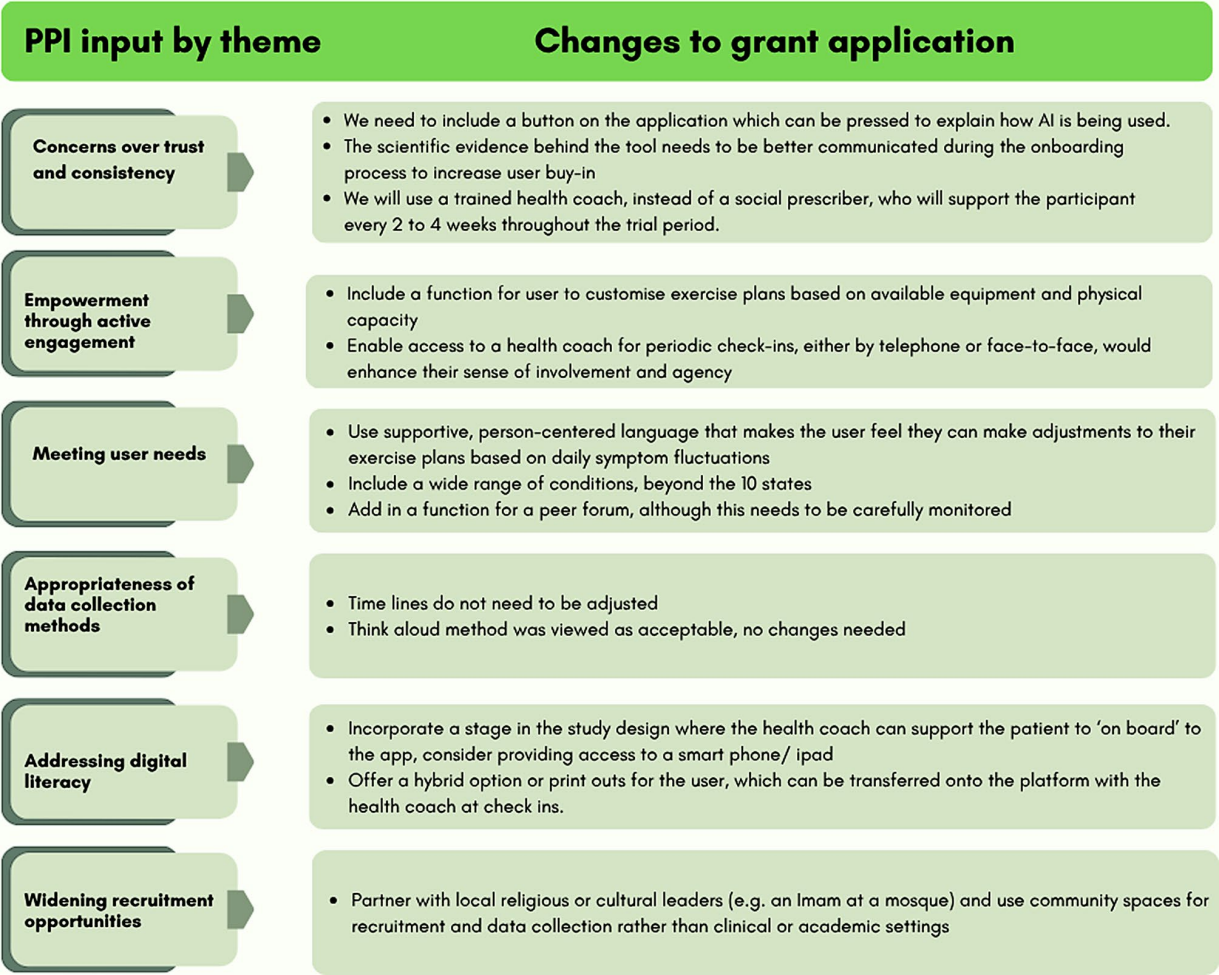
Changed made to grant application based on PPI input

Combined feedback from the narrative synthesis of Activty 1 and 2, and the changes made to the grant application on the basis of the PPI input is shown in Fig. 3.

### Stage 2: Forming a lived experience group

**Fig. 4:**
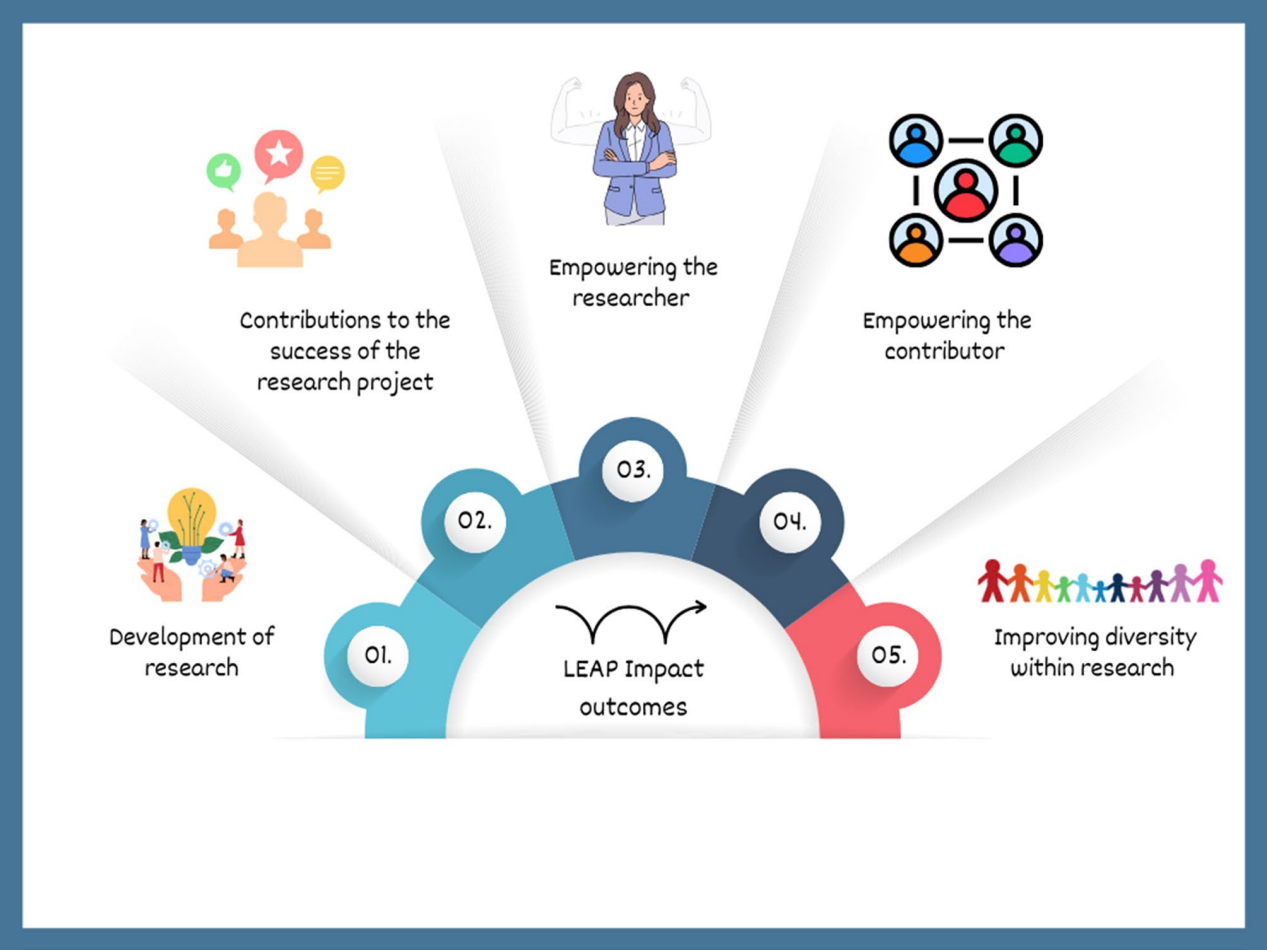
Key impact outcomes of the LEAP

The group determined specific roles, including a chair and vice chair, and co-produced a vision for the list of key functions and impact outcomes of the group, as shown in Fig. 4, influenced by lived experience and examples from other research centres. A full breakdown of anticipated impact can be seen in Appendix 1.

### Stage 3: Writing an initial grant proposal and gathering feedback from LEAP participants

The LEAP group were provided with the draft grant for initial comments. Feedback received was positive and no changes to the design were requested. However, it was suggested we needed greater clarity over who various stakeholders within the grant would be. Concepts such as the ‘think aloud’ method and LifeGuide also needed to be defined more clearly in the grant application so the wider audience could access all the information neceessary.

### Stage 4: Impact assessment

Impact is assessed across the previously mentioned criteria; PPI change log, PPI contributor feedback, contextual and process factors, and the UK standards for public involvement alignment review.

#### PPI change log

Initial PPI group feedback and contributions from the LEAP had a direct influence on the framing of the proposed research, as documented in Table 3, the PPI change log. In response to feedback, the recruitment strategy was revised to prioritise community-based engagement and outreach, including provision of in-person interviews to improve accessibility. Regarding the use of the think-aloud studies, participants emphasised the importance of transparency regarding how the platform communicates its use of AI, and the scientific rationale behind its recommendations. As a result, the study design was updated to include targeted evaluation of users’ understanding of these elements. To support individuals with low digital literacy, the team committed to working collaboratively with users and informal carers to develop an alternative, non-digital access pathway. This aimed to ensure equitable use of the platform, even among those without access to digital devices. In addition, standardised health coach provision was considered for use in the prototype testing phase. The earlier plan to rely on social prescribing teams was revised, following feedback highlighting inconsistencies in access and delivery across primary care settings.

**Table 3:** PPI change log

Feedback	Level of agreement across the group	How can this be incorporated into the grant proposal?
The video needs to be more inclusive of the types of people who have MLTC, other ages and ethnicities need to be involved, and it needs to sound less like a product promotion tool.	Medium – not everyone commented on this statement, and it has since been suggested by one participant that the video may well be representative.	The script needs to be rewritten using feedback from the LEAP so that the aim of the video – which is to explain our research concept to the public – is clear. We need to evaluate the inclusivity of our images.
The use of AI within the tool needs to be more clearly explained to the user	High – no one disagreed	We need to include a button on the application which can be pressed to explain how AI is being used.
The health coach is important, but social prescribers are not available for everyone and we need consistency	High – no one disagreed	We will use a trained health coach, instead of a social prescriber, who will support the participant every 2 to 4 weeks throughout the trial period.
We need to build trust in the exercise recommendations, so people with MLTC feel confident that what they are being asked to do is safe	Medium – some people felt this was more important to their conditions than others	The scientific evidence behind the tool needs to be better communicated during the onboarding process to increase user buy-in.
The use of a community forum could be helpful for users to support each other, but this needs to be carefully managed	Medium – all people but one felt this option was important	The tool should include a section which guides the user to charity websites that have forums for the different conditions, so that community support could be received there, as those platforms are moderated.
Recruitment needs to be taken into the communities rather than expecting them to contact us in response to a research call	High – all people felt this was key to avoid exclusion	We will embed an inclusive recruitment strategy into our work packages that incorporates going in to visit a wide range of community groups across underrepresented populations to build up connections with members of these groups. We will offer in person think aloud studies, within community settings, as well as an online option.
We need to develop a workstream to support those with limited digital literacy use and/or access the tool	High – all people felt this was important	We will create a digital inclusion strategy which is built across the whole work package, to ensure there is an effective alternative provision.
We need to establish who our stakeholder group is across the different work packages,	High – all people who commented felt this was important.	We will rewrite our stakeholder sections within the grant to offer more clarity over who will be recruited and why.

#### PPI contributor feedback

Following the workshop event, four out of seven participants completed the post-event feedback form (see Table 4), and this indicated that the activities were generally well received. One participant noted difficulty using physical props due to their health condition, highlighting the need to consider activity accessibility needs beyond verbal communication in future workshop planning. Several participants appreciated the person-centred nature of the session, commenting that it felt participant-led rather than researcher-driven. Two individuals sent additional commentaries to the researcher:

*Yesterday’s event was wonderful. Compared to other PPI events where the presenters spend 90% of the time making presentations and only allow Q &A at the end of the event and sometimes all the questions don’t get answered. You allow presentations by PPI led and cross discussions among the participants brings out many new features. I enjoy taking part in your events.* (Participant 1).

*I wanted to say a number of things in this email. Firstly*,* what an enjoyable and interesting morning! You both made it very easy to be involved*,* with a relaxed atmosphere yet things were nicely kept on track. Secondly*,* what a brilliant idea! Vitova has the ingredients to be a huge success*,* used in the right way and with the right support. I can’t believe something like this hasn’t been thought of before and think it’s incredibly exciting.* (Participant 6).

However, there were also observations that some participants dominated the discussion:

*Some members of the group had perhaps rather more to say than others (always the case) although I feel one individual may have occasionally prevented others’ contributions (always hard to judge and moderate).* (Participant 6).

A suggestion was made to provide written response opportunities (e.g. writing on panels before speaking), to help balance participation and ensure all voices were equally heard. One participant also felt there was too much content for one workshop session.

The reimbursement process was valued, though one participant suggested that providing a gift voucher only—rather than travel expense reimbursement—might be preferable for those travelling longer distances. It was also recommended that future sessions include an earlier comfort break.

Would have preferred if it had started later given travelling distance. Definitely needed a 10–15 break half-way through. Uncertain when it had ended. The props were unsuitable for me with my rheumatoid arthritis. Not so keen on the goody bag (Participant 7).

**Table 4:** Evaluation of stage 1 - initial gathering of PPI and LEAP feedback

Participant ID	Overall reflections on your experience at the workshop	I felt as though my opinions were heard	I understood the role that I was playing in the research process	The event was designed in a way that I felt able to fully participate	I liked the choice of venue	How we could have improved the event?	Would you be interested in becoming a member of the lived experience research panel (LEAP) for this project
3	Very interesting subject matter and a good group to review how it’d be presented. Some members of the group had perhaps rather more to say than others (always the case) although I feel one individual may have occasionally prevented others’ contributions (always hard to judge and moderate).	5	5	5	5	It was brilliant and very enjoyable - possibly see above re the difficult task of moderating.	Yes
7	Interesting discussions, good to hear about latest research ideas and meet other patients face-to-face	5	5	4	3	Would have preferred if it had started later given travelling distance. Definitely needed a 10–15 break half-way through. Uncertain when it had ended. The props were unsuitable for me with my rheumatoid arthritis. Not so keen on the goody bag as I don’t like chocolates and scented candles - the scented candle was also heavy for me to carry around for the rest of the day, would have preferred an extra £10 on the gift card instead.	Maybe
1	Excellent	5	5	5	5	Please continue in the same unique total participatory involvement. I like it very much. I would not change any format.	Yes
4	Very interesting but a tried to cram too much into the allocated time	5	5	5	5	Allocate more time	Yes

*5 = strongly agree 4 = slightly agree 3 = neither agree nor disagree 2 = slightly disagree 1 = strongly disagree

**Fig. 5:**
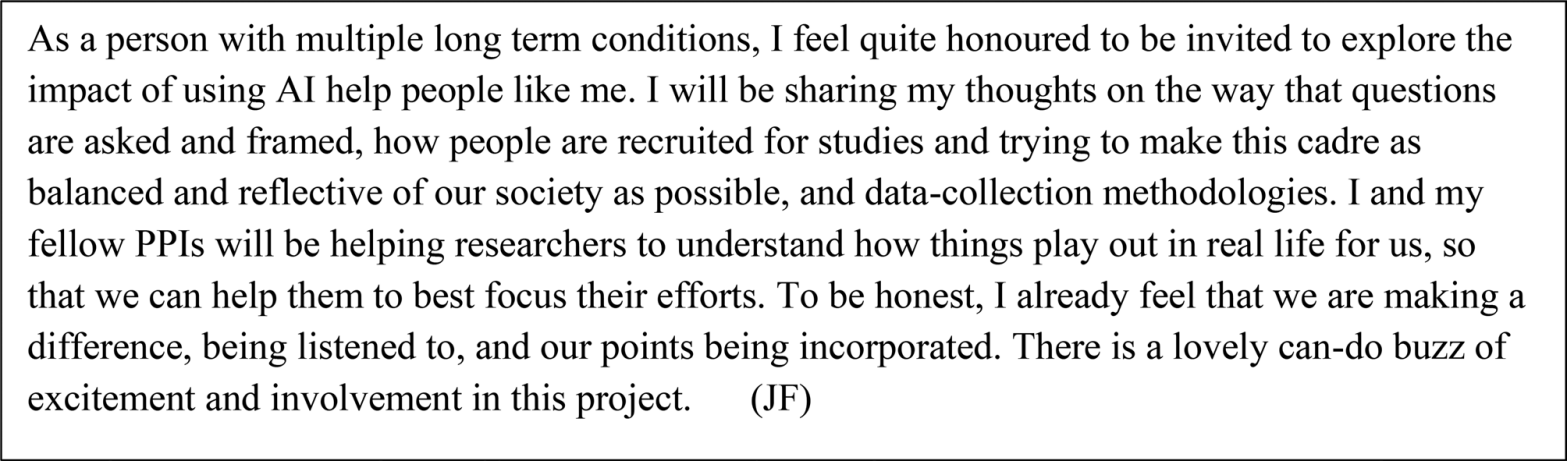
Impact statement from Chair of the LEAP

When asked for feedback of stage 2 – formation of the LEAP, members stated that they found the experience interesting, although it was noted that an appropriate amount of notice for meetings should be provided, and to allow sufficient time to provide feedback on documents. If successful in the grant application, the group have agreed to meet once a month online, with a 5-day turn around for commentary on documents. The chair of the LEAP (JF) has provided the following impact statement (Fig. 5):

#### Wider contextual factors influencing involvement

Several contextual factors influenced participation and inclusivity. Holding the PPI event in person excluded some due to travel or health issues, and others withdrew due to symptom flare-ups. Promotion of the PPI event through a local university PPI network likely skewed attendance toward those already engaged with research or academia. We did try to broaden representation by inviting individuals with chronic conditions not typically involved in PPI. However, all attendees were middle-aged or older, professionally experienced, and highly educated, limiting diversity. One participant recommended promoting future events in community settings like GP waiting rooms to reach less-engaged individuals.

Researcher openness also shaped outcomes; setting aside the need to defend research design and being receptive to public feedback was vital. Our commitment to documenting and acting on this input added transparency and helped the team genuinely incorporate public perspectives—even when they challenged our assumptions—into the grant design.

#### UK standards alignment review [18]

Whilst the UK standards alignment review does not provide a formal process for assessing PPI against the six values, it advocates a reflective approach as outlined in Table 5. The research team discussed the adopted PPI framework across all six values, aided by reflective questions provided by the NIHR. Whilst we are confident that each standard was met, we identified the need to embed more inclusive recruitment strategies in the PPI process going forward, and consider accessibility for practical workshop activities against user needs.

**Table 5:** An assessment of the PPI framework against the UK standards alignment review (NIHR 2019)

Core Value	Evaluation
Inclusive opportunities	• We recruited people affected by MLTC and carers. • We considered barriers to involvement, providing additional payment for travel and reimbursed contributors for their time. • We received positive feedback on our communication, although the video was criticised for not being diverse enough. • We have been transparent in the fairness of our processes, and this helped us to identify the need for more diverse recruitment methods.
Working together	• The LEAP co-produced the purpose and function of the group, creating 5 key impact areas. • We recorded all outcomes from the PPI process and considered the practical applications for working with contributors. This highlighted that some of our workshop activities need to be altered due to difficulties with joint pain. • The workshop activities were not planned with a PPI representative, but all future LEAP meetings will be chaired by a lived experience contributor. • We have documented all contributions by each LEAP member and workshop attendee.
Support and Learning	• The research team has expertise in PPI and engagement events, and this has been used to strengthen the culture of inclusivity across the research team. • LEAP members have been asked to consider their training needs, and we have been transparent in how contributors can (i) be involved across the research process (ii) access additional support needed. • We need to extend our PPI reach to under researched populations, and future work will seek to strengthen our contextual understanding of how to achieve this by seeking expertise from members of these communities as well as the LEAP.
Communications	• We are sharing our PPI process through wider dissemination activities including publications, a video and the release of a plain English summary. • The PPI change log captures the feedback from PPI and the changes that occurred as a direct result of this, which is shared with the LEAP at monthly meetings.
Impact	• The LEAP has co-produced our impact outcomes. • We have made our impact assessment structure transparent across our entire PPI process to the public.
Governance	• All data is anonymised and stored on a secure platform within Southampton University. • We have discussed resources including costs, training and time to conduct our PPI framework. • All LEAP meetings are documented through the use of Co-pilot minute generation to provide accountability of involvement through the project. These are validated within the LEAP to ensure all voices have been represented.

## Discussion

This paper has described a novel framework for involving patients and members of the public in the development of a research grant focused on AI-supported exercise prescriptions for individuals living with chronic illness and has assessed the impact of this. Rather than reporting on a completed study, the aim was to document how PPI was embedded across all stages of grant development—from early idea generation to the preparation of the final proposal, and to meaningful overall assessment of impact. We found that the 4-stage framework was well received by contributors, and by taking a robust evaluation approach, we have been able to critically analyse the impact of PPI on our grant development process. This paper provides a framework which can be adopted by other researchers to enhance the integrity of PPI across the research life cycle, reframing the tokenistic approach that is often taken within the grant development process.

### Comparison to previous literature

In line with current literature, our findings demonstrates that PPI can, and *should*, take many forms across the research cycle [19–21]. A recent review of digital tools for the self-management of MLTC did not find any inclusion of a low digital literacy intervention arm, with inclusion criteria requiring the participant to already have access to the internet and digital tools [22]. The need to consider a hybrid approach for those with low digital literacy, as well as those digitally excluded emerged from the feedback, which we have now built into our work packages. Shen et al.(2017) suggest that research which engages PPI leads to more meaningful and socio-culturally appropriate research designs [23]. The changes made to our research design are in line with this position, highlighting the importance of incorporating PPI at the start of the grant development process, where changes can still be made to the research design. In our consideration of wider contextual factors which may have influenced the impact of our PPI, we noted the need to rethink our approach to recruiting from under-represented communities, an issue that has been extensively reported in the literature [24]. Whilst we did have representation from White and Asian communities, other ethnic groups were not represented, and all LEAP members have a high level of education and professional backgrounds, demonstrating that representation within PPI remains an issue. This is in line with feedback from a recent paper by Gathani et al., which highlighted the need to build in a realistic time frame for recruiting for ethnic minority groups, and the importance of being transparent with potential contributors about the researchers need and activities that will be incorporated into the PPI [25]. A key feedback point from our PPI contributors was the suggestion of recruiting from within global majority communities, a learning point which will improve the appropriateness of our sampling strategy in the future. Previous research shows a lack of clarity around how to carry out and report PPI in the grant development process [2, 3, 12], our research provides evidence that a clearly operationalised framework can be applied to guide the academic community through this process. This adds to existing literature by Chudyk et al., who identified seven different ways that PPI contributors can be involved in research [26], by providing guidance on how to effectively plan a clear PPI delivery and impact measurement strategy within which these activities could be encompassed.

### Strengths and limitations

Our framework effectively facilitated and evaluated early-stage PPI, with in-person group setting enabling spontaneous idea generation, rich dialogue, and exploration of diverse experiences. Compared to previous one-to-one interviews or usability testing, the group dynamic encouraged more collaborative reflection. A well-structured session with icebreakers and clear, facilitated activities fostered rapport and a supportive environment. Although the in-person format may have limited participation for those with mobility issues, fluctuating symptoms, or geographic constraints. The workshop also surfaced key barriers to early PPI, including financial and logistical challenges; however, participants appreciated the use of NIHR-aligned reimbursement procedures. Further valuable feedback emerged, such as suggestions to replace clinical language like “compliant,” incorporate nutrition components and clinical test result support, as well as continuing to prioritise accessible, inclusive design. Participants expressed enthusiasm for continued involvement through the LEAP, viewing it as a meaningful opportunity to shape research and development. Assessing impact across four key areas provided a holistic view of the PPI process. Particularly by reflecting on the broader contextual and process factors that shape PPI effectiveness, and enhance the researchers’ understanding of patient and public perspectives. In addition, a strength of our approach were the bidirectional outcomes for the contributors, who expressed during the workshop and subsequent LEAP meetings, that they felt a sense of empowerment and that they were genuinely ‘being heard’.

However, several limitations remain. Recruitment relied heavily on existing contacts or the Oxford PPI network, which limited diversity. Future efforts should broaden outreach by partnering with community organisations serving marginalised populations, or approaching community leaders and trusted figures to help disseminate event information to those who may lack access to PPI opportunities. This is reflected in our current LEAP composition: four white members, one Asian member, and a white coordinator (LS). The group are also educated to a high standard, which lowers representation of the wider population. Future work will include assessing the impact of this limited diversity and implementing a purposeful strategy to engage underrepresented communities, such as engaging with community leaders as mentioned above, or hosting workshops in locations often frequented by under-represented communities.

Our framework would be strengthened by a wider consideration of appropriate budgeting of PPI in the grant writing process, and across the research life cycle. Simoni et al., (2023) provide clear guidance through a worked example, from the PPI budgeting in the Asthma UK Centre for Applied Research, which could be incorporated into the framework, with better attention being given to training costs for PPI, costs for running the activities, capacity costing for staff to manage the PPI [27].

Other research which has explored the use of PPI has provided a deeper exploration of the impact of power sharing, where the dynamics between the researchers and contributors needs to be acknowledged [28]. In our study, researchers’ own positionality on the use of AI derived tools for PA must be acknowledged; JK who supported with the facilitation of the workshop is the CEO of VITOVA, an AI derived tool for PA interventions, and the researcher (LS) who led the session and subsequent LEAP meetings has a favourable attitude towards the use of these tools; it may be the case that contributors experienced feelings of being swayed positively towards the grant concept. The themes which were developed from our narrative synthesis were practically driven, that is, they were gathered with the aim of shaping our grant application. However, the use of colour cards for contributors to express their opinions yielding a mix of colours from them shows that contributors were able to provide their genuine feelings about the concept, and feedback being driven by the contributors rather than the facilitators should have also lessened the likelihood of bias.

Lastly, a further limitation of this case study refers to the inequity which likely occurred due to the lack of form training for some of the contributors. As our LEAP group continues to develop formal PPI training will be provided to standardise the level of experience across the team. Although equity and accessibility were considered during design, we did not formally assess potential differential impacts using an established equity framework. Future work should explicitly examine how digital and AI-based elements may differentially affect underserved or marginalised groups to ensure equitable benefit, so that training needs can be met, a finding echoed by Birch et al., (2020) who provide recommendations for how to formalise this process within PPI work [29] and Foster et al., (2024) who have shown the need for provided sufficient funds to meaningfully engage with contributors across the grant development process, and, after the project has ended for meaningful dissemination [30].

## Conclusion

By applying the novel PPI framework proposed here, consisting of PPI group feedback, LEAP creation, ongoing LEAP involvement in grant design, and ongoing impact assessment, contributors meaningfully shaped grant research questions and design, enhancing the relevance, accessibility, and inclusivity of the final proposal. This paper introduces a practical and adaptable framework to support researchers in embedding high-quality PPI into grant development, ultimately leading to more impactful and patient-centred outcomes. We emphasise the importance of recognising and addressing the broader contextual factors—such as recruitment pathways and structural barriers—that can limit meaningful involvement, particularly from underrepresented communities. Targeted, community-based outreach is essential to ensure PPI contributes to reducing health inequalities rather than reinforcing them. Finally, we demonstrate that PPI impact can be evaluated systematically across multiple domains, and we encourage other research teams to adopt similar approaches to strengthen the visibility, credibility, and influence of public involvement throughout the research lifecycle.

## Appendix 1

**Table 3:** A co-produced vision for the function and impact of the LEAP

Key function	Anticipated impact
Development of research	Give feedback on the research question, plan, recruitment, and data collection early in the grant process to ensure the research is relevant, ethical, and effective.”
Contributions to the success of the research project	provide feedback throughout the research, including recruitment, results, writing the paper, and developing dissemination materials, enhancing knowledge mobilisation impact
Empowering the researcher	Greater PPI involvement will boost researchers’ confidence in conducting PPI, increase awareness of patient/carer experiences, improve engagement with underrepresented groups, and enhance public involvement in research
Empowering the contributor	Members will feel valued and empowered to influence health and social care, gaining confidence in accessing research. This will encourage greater participation from harder-to-reach groups in future studies
Improving diversity within research	PPI contributors will bring diverse lived experiences, helping researchers design more inclusive studies that reflect underrepresented populations

## Electronic supplementary material

Below is the link to the electronic supplementary material.

Supplementary Material 1

## Data Availability

No datasets were generated or analysed during the current study.
