# Supplementary figures and images for: A novel framework for operationalising patient and public involvement: lessons from designing an AI-informed exercise prescription grant
Source: Res Involv Engagem. 2025 Oct 29;11:130. doi: 10.1186/s40900-025-00801-4 (PMC12574169; doi:10.1186/s40900-025-00801-4)

Supplementary Fig. 1: Advertisement for PPI Session.


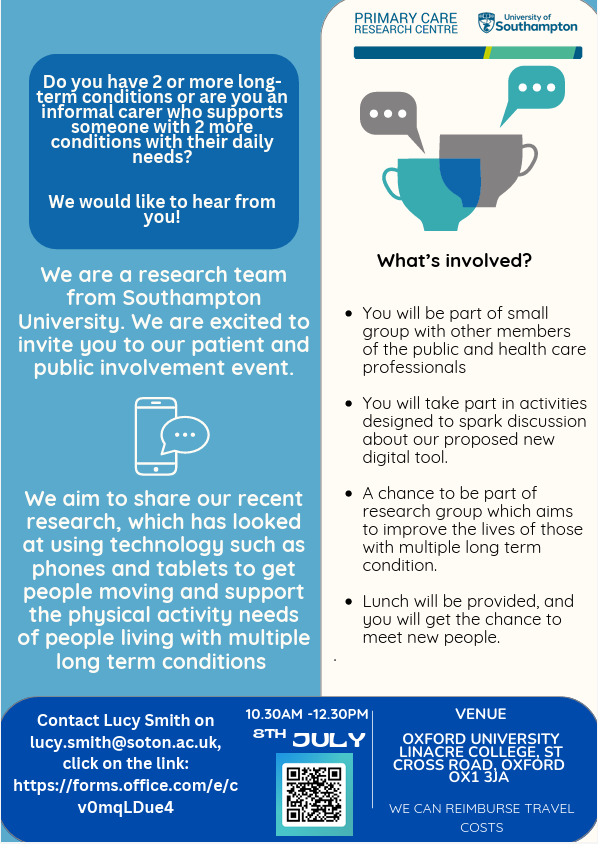

Supplement: Supplementary file 1 — Supplementary Material 1 [file 40900_2025_801_MOESM1_ESM.docx]
